# Grid-based transcutaneous spinal cord stimulation: probing neuromodulatory effect in spinal flexion reflex circuits

**DOI:** 10.1088/1741-2552/adc6bd

**Published:** 2025-04-07

**Authors:** Hyungtaek Kim, Subaryani Soedirdjo, Yu-Chen Chung, Kathryn Gray, Sofia Rita Fernandes, Yasin Y Dhaher

**Affiliations:** 1 Department of Physical Medicine and Rehabilitation, University of Texas Southwestern Medical Center, 5323 Harry Hines Blvd, Dallas, TX 75390, United States of America; 2 Department of Bioengineering, University of Texas at Dallas, 800 W Campbell Rd, Richardson, TX 75080, United States of America; 3 Instituto de Biofísica e Engenharia Biomédica, Faculdade de Ciências, Universidade de Lisboa, Lisbon, Portugal; 4 Peter O’Donnell Jr. Brain Institute, University of Texas Southwestern Medical Center, 5323 Harry Hines Blvd, Dallas, TX 75390, United States of America

**Keywords:** flexion reflex, *H*-reflex, spinal excitability, transcutaneous spinal cord stimulation, multi-electrodes tSCS

## Abstract

*Objective.* Non-invasive spinal stimulation has the potential to modulate spinal excitability. This study explored the modulatory capacity of sub-motor grid-based transcutaneous spinal cord stimulation (tSCS) applied to the lumbar spinal cord in neurologically intact participants. Our objective was to examine the effect of grid spinal stimulation on polysynaptic reflex pathways involving motoneurons and interneurons likely activated by A*β*/*δ* fiber-mediated cutaneous afferents. *Approach.* Stimulation was delivered using two grid electrode montages, generating a net electric field in transverse or diagonal directions. We administered tSCS with the center of the grid aligned with the T10–T11 spinous process. Participants were seated for the 20 min stimulation duration. At 30 min after the cessation of spinal stimulation, we examined neuromodulatory effects on spinal circuit excitability in the tibialis anterior muscle by employing the classical flexion reflex paradigms. Additionally, we evaluated spinal motoneuron excitability using the *H*-reflex paradigm in the soleus muscle to explore the differential effects of tSCS on the polysynaptic versus monosynaptic reflex pathway and to test the spatial extent of the grid stimulation. *Main results.* Our findings indicated significant neuromodulatory effects on the flexion reflex, resulting in a net inhibitory effect, regardless of the grid electrode montages. Our data further indicated that the flexion reflex duration was significantly shortened only by the diagonal montage. *Significance.* Our results suggest that grid-based tSCS may specifically modulate spinal activities associated with polysynaptic flexion reflex pathways, with the potential for grid-specific targeted neuromodulation.

**Abbreviations**
tSCS: transcutaneous spinal cord stimulation
TA: tibialis anterior
Sol: Soleus
AUC: area under curve
ROR*α*/*β*: Retinoid-related orphan receptor alpha/beta
Pre: baseline
Post: 30 min after spinal stimulation offset

## 1. Introduction

Over the past decade, several spinal cord stimulation techniques have demonstrated their effectiveness in neuromodulating spinal circuits, offering potential improvements in voluntary movement for both animals [1, 2] and humans [3–5] with spinal cord injury (SCI) and stroke. In particular, implanted epidural spinal stimulation (ES) has shown its ability to alter the state of spinal motor circuits, restoring the capacity for independent standing [3, 6, 7] and locomotion [3, 4], even in individuals with motor-complete SCI when paired with locomotor training [7–9]. While these findings are encouraging, the implanted stimulation technique is invasive and involves expensive surgical procedures for electrode placement.

Promising outcomes have been observed with non-invasive tSCS. Studies have reported enhanced voluntary lower extremity function, including standing and locomotion [10, 11], and reduced spasticity [12, 13] when tSCS is paired with traditional rehabilitation training. Although this method has yet to match the efficacy of ES [4, 9, 14], tSCS offers distinct advantages, particularly for patients who may not be suitable for or wish to avoid surgical procedures due to the associated risks and side effects, costs, and extended recovery periods.

However, as with any electrical stimulation modality, a key factor for tSCS efficacy is the spatial extent of the induced electric field. Currently, electrode placement for tSCS consists of positioning an electrode over the targeted spinal cord region for modulation. In contrast, the return electrode is placed in a spatially distant area with a location that ensures adequate current delivery at the site of interest. Computational modeling studies indicate that employing this bipolar scheme results in a diffused electric field that extends beyond the intended spinal segment rather than being confined directly beneath the electrode. These in-silico examinations imply that the bipolar electrode configurations used in current tSCS studies may not have delivered the maximum or optimal electric field to the intended target segments. Thus, a focal stimulation with control over the direction of the electrical field may improve motor recruitment.

Several potential mechanisms have been proposed to explain the effects of spinal stimulation, including stimulation of dorsal root afferents and monosynaptic activation of motoneurons [15], modifications in motor commands transmitted along the corticospinal pathway [16], and stimulation-induced modulations in the excitability of propriospinal neuron networks and spinal interneurons [17]. The dorsal root afferents play a crucial role in this process by conveying excitatory post-synaptic potentials to the spinal motoneurons via mono- and poly-synaptic connections [15, 18–20]. Other mechanisms may also contribute to the effect of spinal stimulation, including changes in polysynaptic spinal reflexes, likely engaging both motoneurons and interneurons [21, 22]. Indeed, in-silico estimation of the electric field generated in the spinal cord by tSCS suggested that the induced electric field [23] is sufficient to modulate firing patterns of axons within ascending and descending white matter tracts and neurons [24] in the spinal gray matter as well [25]. Clinical studies on SCI patients suggest that tSCS can modulate the excitability of sensory-motor pathways, including interneuronal circuits, during stimulation [26, 27].

While it has been reported that some functional improvements continued to be present for an extended period after the cessation of spinal stimulation [28]. Most existing tSCS paradigms seek to achieve real-time neuromodulation, enabling patients with permanent motor deficits or incomplete paralysis to achieve improved walking abilities [29–31]. However, few have been done to test the potential short-term changes in neuronal excitability post-spinal stimulation [13, 21, 32, 33].

In this study, we investigated the modulatory capacity of a grid-based, sub-motor threshold tSCS applied to the lumbar spinal cord in neurologically intact participants. Our grid design delivered spatially localized stimulation, unlike the classical bipolar stimulation paradigms where electrodes are placed on different parts of the participant’s body (shoulder, abdomen, back, and pelvis). This design was informed in part by our reported computational model of the spinal cord [23]. By locating the cathode and anode nearby, we intended to increase the spatial resolution of the stimulation while ensuring that the maximum intensity of the electric field remained well above the established threshold (0.15 V m^−1^) for effective neuromodulation sufficient to bias or augment ongoing rhythms [23, 34, 35], yet substantially below activation thresholds observed in ES, estimated at around 180 V m^−1^ based on a clinical study [36, 37]. This grid configuration was applied at the T10–T11 spinous processes, overlying the T10–T12/L1 vertebral level, to localize the electric field at the lumbar spinal circuits that projected to the lower-limb muscles. In addition, we employed a stimulation paradigm with two montages that generate a net-applied electric field in the transverse and diagonal directions. These arrangements of anode and cathode were selected as ‘feasibility/proof of concept’ montages, with the model indicating that they lead to discernible variations in both the direction and magnitude of the electric field within the gray matter [23]. We seek to illustrate that reshaping the neurophysiological properties of the spinal circuits can be achieved by rearranging the cathode and anode configuration of the grid.

We aimed to assess the effect of spatially localized spinal stimulation on the excitability of spinal neural circuits, focusing on spinal reflex pathways involving motoneurons and interneurons mediated by A*ß*/*δ* fibers, known to play a role in motor coordination [38, 39]. In a subcohort of participants, we sought to explore the potential modulatory effect of grid stimulation on motoneuron excitability using the *H*-reflex probe in the adjacent non-targeted motoneurons. We examined the neuromodulatory effect 30 min after the offset of the stimulation to assess the short-term effects (plasticity) and exclude any stimulation-induced effects due to temporary membrane polarization of spinal roots or white matter tracts. We employed two grid montages to investigate the influence of electric field direction on the short-term changes in spinal neural excitability. We hypothesize that grid tSCS will induce changes at the circuit level and that the observed change will be grid-montage dependent. This pilot study aims to explore the effects of grid-specific montages on reflex pathways, thereby enhancing our understanding of how spatial variations in the induced electric fields impact spinal neural activity.

## 2. Methods

### 2.1. Study design

**Figure 1.**
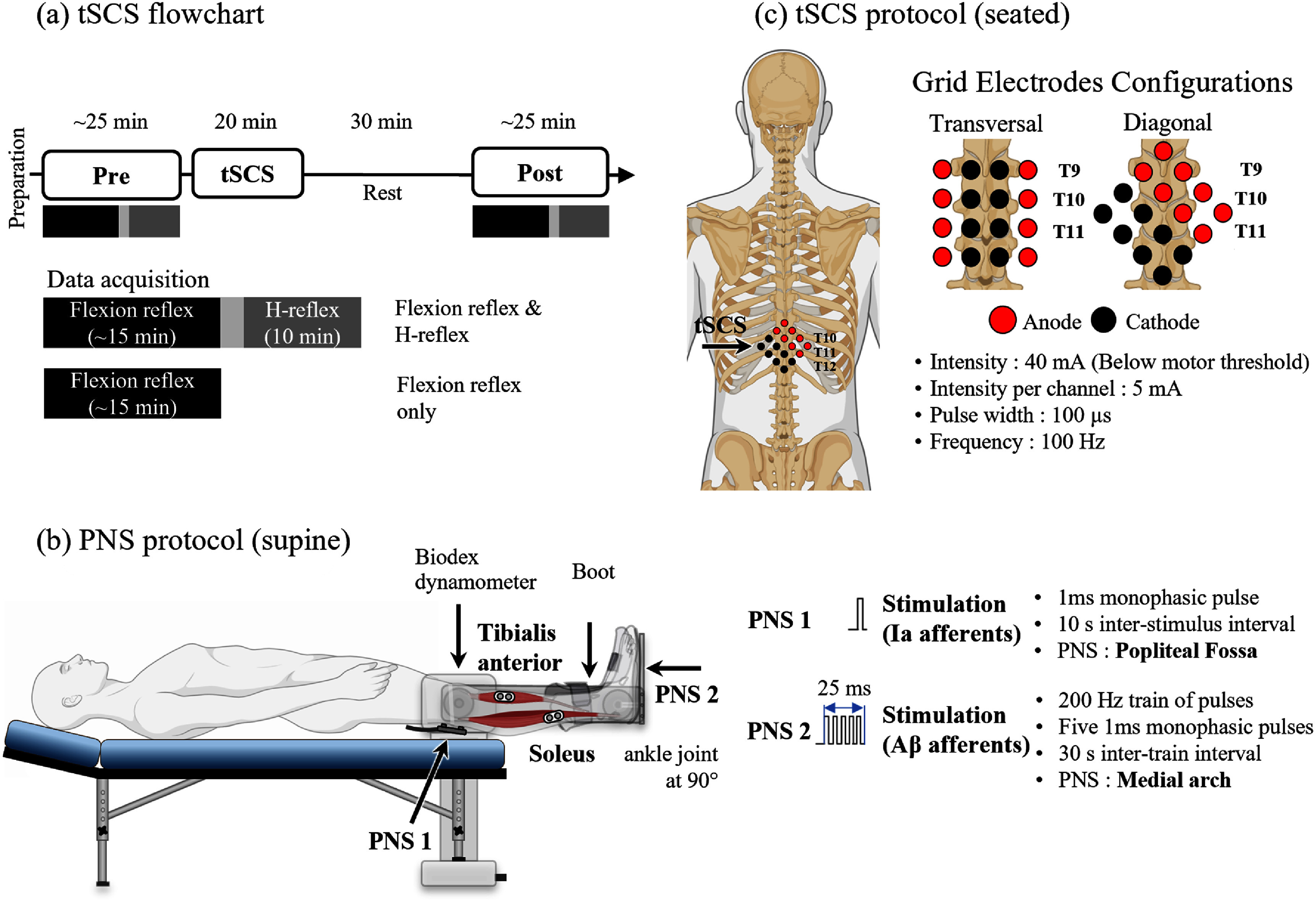
Experimental study design. (a) Flowchart of the full experimental protocol showing data collection before (Pre) and 30 min after (Post) grid tSCS. (b) Illustration of electrode placements for peripheral nerve stimulators (PNS1 and PNS 2) and EMG recordings at right TA and Soleus. (c) Grid placement (left) and configurations for transversal (TRS) and diagonal (DIAG) montages, with anodes shown in red and cathodes in black. Active tSCS was delivered to the spinal cord using a TENS stimulator at a current intensity of 40 mA, frequency of 100 Hz and pulse width of 100 µs for 20 min. In the sham condition, TRS stimulation was applied during the initial 30 s (from 2 mA to 40 mA) and set to 0 mA for the remaining 19.5 min. Created in BioRender. Dhaher, Y. (2025) https://BioRender.com/tyy4rgf.

Eighteen healthy male participants (mean age 28.9 ± 6.36; mean BMI of 24.3 ± 3.33 kg m^−2^) were recruited for the study. One participant was excluded from the flexion reflex analysis due to an inability to elicit a response. Thirteen participants completed two active tSCS sessions and one sham tSCS session to evaluate the placebo effect. Four participants only completed two active sessions due to various reasons, including leg injury, relocation, or scheduling conflicts that prevented them from returning. The flexion reflexes of the right leg were assessed at two time points: before tSCS (Pre) and 30 min after the end of tSCS (Post) (figure 1(a)). The decision to implement a 30 min post-stimulation observation period was based on previous literature [40] to differentiate between immediate effects, such as those induced by temporary depolarization, and short-term changes in spinal neuronal excitability.

A subgroup of seven participants underwent the *H*-reflex procedure following flexion reflex assessment during both active tSCS sessions. Participants were instructed to abstain from caffeinated beverages, alcohol, and excessive exercise for at least 12 h before each session. All participants were blinded to the type of tSCS administered (TRS, DIAG, or Sham).

Each session was scheduled at least four days apart to minimize possible carryover effects. Only male participants were included in this study to eliminate the potential hormonal effects on reflex afferents during the menstrual cycle in females [41–43]. All participants completed the study without experiencing adverse effects such as pain or skin burns. The Institutional Review Board of the UT Southwestern Medical Center approved the study. The study protocol was explained to each participant, and informed consent forms were signed before the first tSCS session.

During the session, participants were in a supine position on the examination table. A boot was attached to the Biodex dynamometer (Biodex Rehabilitation System 3, Shirley, NY), and the right foot was securely placed inside the boot using a Velcro strap, maintaining the ankle at 90° throughout the session (figure 1(b)). Surface electromyograms (EMGs) were recorded using bipolar differential electrodes (Bagnoli DE-2.1; inter-electrode distance 10 mm; contact dimensions 10 × 1 mm; Delsys, Boston, MA), which were placed on the bellies of right TA, peroneus longus (PL), soleus (Sol) muscles. The application of these electrodes followed a preparatory procedure that included removing body hair with a razor and cleaning the skin with an alcohol swab and an abrasive paste. The locations of the electrodes were measured relative to bony landmarks to ensure consistent placement across subsequent sessions. The EMG signals were digitized at 2 kHz using Micro1401, and the acquisition was controlled by Spike2 (Cambridge Electronic Design Ltd, Cambridge, UK).

### 2.2. TA flexion reflex procedure

A total of seventeen participants were tested for the TA flexion reflex. The TA flexion reflex was elicited by delivering electrical stimuli through pre-gelled surface electrodes (K.S.Choi Corp, Los Angeles, USA). The electrodes consisted of a 2 cm diameter anode placed in the medial arch and a 5 × 5 cm cathode positioned in the dorsum of the foot (figure 1(b)). Stimulation was delivered using a constant current stimulator (DS7A, Digitimer, Welwyn Garden City, UK) and consisted of a train of five individual monophasic pulses (1 ms duration, delivered at 200 Hz). The current intensity was incrementally increased by 1 mA until the initial TA EMG activity was observed within a latency window of 60–100 ms after the onset of the constant current train [44]. Latencies around 100 ms have also been reported in the previous study [45], and were occasionally observed in this study. Subsequently, the intensity was decreased using a smaller increment (0.25 mA) to determine the flexion reflex threshold. The threshold was defined as the minimal intensity at which the peak-to-peak reflex amplitudes were above 100 *µ*V two consecutive times. The stimulation intensity for the flexion reflex was set to 120% of the threshold. Participants reported stimulation intensities (5.88 ± 4.3 mA) as non-noxious. Stimulation trains were delivered with random interstimulus intervals between 30 and 40 s to prevent potential habituation effects on the flexion reflex. Each participant received up to 22 stimulations to acquire 10 valid flexion reflex responses (see Method section 2.5), averaging 16 ± 4 stimulations per subject. Both the flexion reflex threshold and the intensity of the stimulation were determined for each time point (Pre and Post) within the session.

### 2.3. Soleus H-reflex procedure

A subset of seven participants from the total cohort was tested for the Sol *H*-reflex. The Sol *H*-reflex was elicited by stimulating the posterior tibial nerve near the popliteal fossa with a bipolar stimulating electrode (10 mm in diameter, 30 mm spacing, anode placed proximally). The placement of the electrode was optimized to target the area that yielded a greater EMG response in the Sol muscle compared to the PL muscle. Stimulation was delivered via a constant current stimulator (DS7A, Digitimer, Welwyn Garden City, UK), which delivered a 1 ms rectangular pulse (figure 1(b)). The interval between stimulation was at least 10 s to avoid possible post-activation depression [46]. In both time points (Pre, Post), a Sol *H*-reflex and *M*-wave recruitment curve was obtained by progressively increasing the stimulus intensity in 1 mA increments until the *M*-wave reached maximal peak-to-peak amplitudes. The intensity required to elicit the maximal *H*-reflex and *M*-wave was then determined from the recruitment curve. The maximal peak-to-peak response of the *H*-reflex and *M*-wave were collected three to five times to ensure the accuracy and reliability of the measurements. In cases where subjects found the stimulation too intense, only three peak *M*-wave responses were collected. Additionally, any *H*-reflex trials in which the subject moved during stimulation were excluded from the analysis. The flexion reflex and *H*-reflex procedures were conducted while participants were at rest. Only the right foot was assessed as the primary focus was investigating spinal reflex responses to two different electric field spatial distributions.

### 2.4. tSCS

tSCS was administered using a grid of electrodes and a transcutaneous electrical nerve stimulation (TENS) unit (Intensity 10, Current Solutions LLC, Austin, TX). The grid of stimulating electrodes was fabricated using pre-gelled electrodes with a diameter of 0.5 inches and an inter-electrode distance of 0.75 inches, comprising eight pairs of anodes and cathodes (figure 1(c)). The T10 and T11 spinous processes were identified by palpation, and the central point of the grid electrode was aligned with the midpoint between the T10–T11 spinous processes. The grid electrode was spaced along T10–L1 vertebrae, which covered the L1 to L5 cord segments [37], based on the anatomical relationship between the spinous process and vertebrae [47].

The stimulation protocol consisted of 0.1 ms biphasic pulses delivered at 100 Hz. A total of 40 mA was supplied to the splitter box and then distributed across eight channels in parallel, resulting in a delivery of 5 mA per channel. While our choice of tSCS parameters differs from some common practices in tSCS research to improve lower limb functions, the literature presents a wide range of stimulation parameters. These include studies where TENS was applied to reduce back pain [48] and where tSCS was used to alleviate spasticity in SCI patients [12, 13]. Notably, research has shown that 100 Hz stimulation resulted in greater spasticity reduction in SCI compared to lower frequencies [49], which was further supported by a meta-analysis study indicating the superiority of 100 Hz TENS over frequencies below 50 Hz [50].

In our study, the stimulation intensity gradually increased from 2 to 40 mA over 1 min and maintained at 40 mA for 20 min. Our choice of the total current was guided by our recent high-fidelity finite element model, indicating that the 40 mA intensity is sufficient to modulate the firing properties of axons within ascending and descending white matter tracts and neurons in the spinal gray matter [24] as well for the two selected montages in this study [51].

Two grid montages were employed, generating a net electric field that propagated along the transverse plane (TRS) and diagonal plane (DIAG) of the spinal column (figure 1(c)). The TRS grid montage was used for the sham stimulation, with an initial intensity increase from 2 mA to 40 mA over 30 s, followed by 19.5 min of no stimulation. Participants were seated during tSCS.

**Figure 2.**
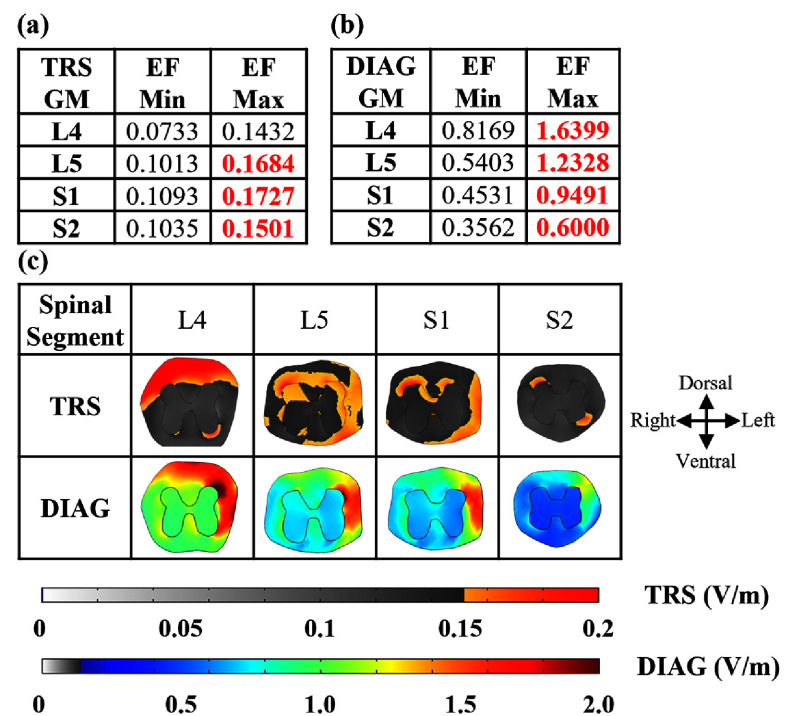
Electric field induced by the tSCS grid predicted by a computer simulation. (a) Minimum and maximum values of the electric field (EF) magnitude in the gray matter (GM) of each spinal segment predicted for TRS (a) and for DIAG (b) montages, with peak values > 0.15 V m^−1^ highlighted in red. (c) Colormaps of EF magnitude distribution of L4 to S2 spinal segments predicted for TRS and DIAG montages, with the corresponding color scales on the right; areas with values < 0.15 V m^−1^ are displayed in a grayscale.

A computer simulation was conducted following the computational framework outlined in Fernandes *et al* [23]—indicated that our grid tSCS configuration is predicted to induce a peak electric field exceeding 0.15 V m^−1^ in L4 to L5 and S1 to S2 segments (figures 2(a)–(c); supplementary tables 1(a) and (b)). These segments correspond to the neural circuits innervating the TA and Sol muscles [37]. This threshold has been previously demonstrated to modulate spike timing *in vitro* [34] and neurons in the human brain [35].

In our prior in-silico simulations, we showed that a maximum current density of 0.802 mA cm^−2^ in the spinal cord and 2.94 mA cm^−2^ in the skin was achieved when using the stimulation parameters employed in the active stimulation conditions [52]. The induced current densities were well below the magnitude that could cause tissue or neural damage (25 mA cm^−2^) [53]. Although none of the subjects reported pain, some study participants reported a slight itching sensation during tSCS, but this sensation subsided within a few minutes after the onset of the stimulation.

### 2.5. Data and statistical analysis

The EMG responses were processed offline using MATLAB software (2022b, MathWorks Inc., MA, United States). Following DC offset removal, EMG responses were filtered by a 4th-order zero-phase Butterworth band-pass filter (20–450 Hz). The power line interference was attenuated using a 2nd order zero-phase Butterworth notch filter (60 Hz).

**Figure 3.**
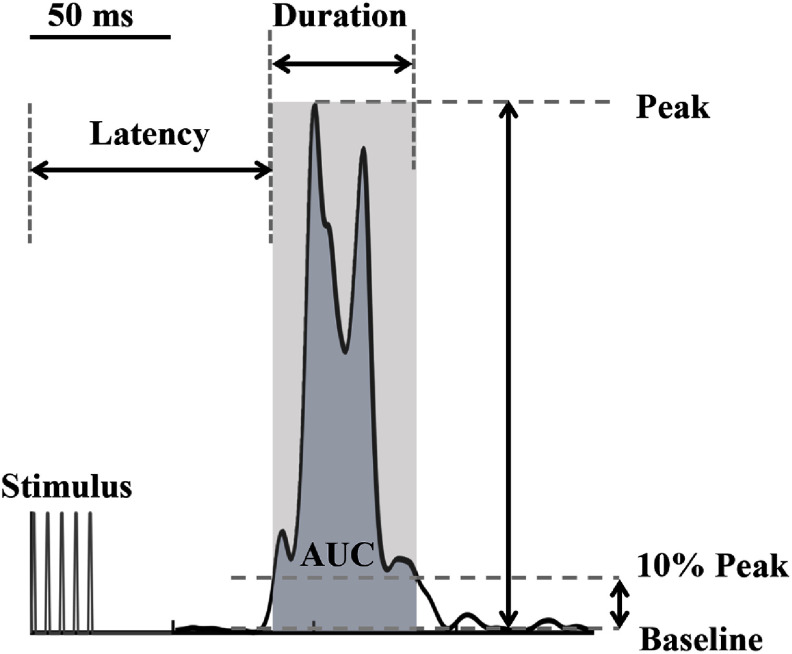
TA flexion reflex response rectified EMG envelope of TA flexion reflex. The onset and offset of the response were defined as the start and end points of the envelope exceeding 10% of the peak amplitude. AUC (area under the curve from onset to offset), duration (time from onset to offset), and latency (time to onset) were calculated based on these parameters.

Three parameters used to characterize the TA flexion reflex were the latency, the duration, and the AUC. The EMG response of the TA muscle was rectified for analysis of the flexion reflex. The envelope of the TA EMG was identified using a 4th-order zero-phase Butterworth low pass filter (100 Hz). The onset and offset of the flexion reflex were determined as the start and end points where the envelope of the TA EMG exceeded 10% of the peak amplitude (figure 3). Valid flexion reflexes were captured with onset latencies ranging from 60 to approximately 100 ms following stimulation onset [39, 44]. The reflex latency was then calculated from the onset of the stimulus. The flexion reflex’s AUC was calculated within the onset and offset window. Given that the amplitude and duration were increased with the higher stimulation intensity, AUC was subsequently normalized to the stimulation intensity [45]. The flexion reflex data were excluded if TA muscle pre-activation was detected. Muscle pre-activation was considered present when the average envelope of the TA EMG during the 100 ms window before stimulation onset exceeded the average TA background EMG by three standard deviations. Background EMG was calculated during the 100 ms period at the beginning of each data acquisition period [39].

Peak-to-peak amplitudes of the *H*-reflex (*H*_max_) and *M*-waves (*M*_max_) in the Sol muscle were identified from non-rectified EMG. We averaged three to five maximal *H*-reflexes and maximal *M*-waves. We normalized the averaged maximal H-reflex to the averaged maximal *M*-wave (*H*_max_/*M*_max_) to compare spinal excitability between different time points (Pre, Post) and between different sessions of tSCS.

Statistical analysis was performed using IBM SPSS software (IBM, Armonk, NY). The flexion reflex and *H*-reflex changes between Pre and Post were assessed. We employed a linear mixed-effects model to examine the effects of the two time points (Pre and Post) on the outcome measures of TA flexion reflex (AUC, latency, and duration) and Sol *H*-reflex (*H*_max_/*M*_max_). Each outcome measure was the dependent variable, with ‘subject ID’, and ‘time point’ as the independent variable. To account for skewness in the data, a log transformation was applied to the outcome measure before analysis. The variable ‘time point’ was treated as a fixed effect to examine short-term changes (Pre vs Post). The variable ‘subject ID’ was included as a random intercept to account for inter-individual variability in baseline responses. Additionally, ‘time point’ was modeled as a random slope, allowing for the effect of ‘time point’ to vary across subjects, capturing individual differences in how subjects respond to grid tSCS. The statistical model parameters were estimated using the Restricted Maximum Likelihood approach [54] to obtain unbiased covariance parameters, and the Satterthwaite approximation [55] was employed for more accurate inference. Fixed effects were examined using Type 3 tests. The estimates of marginal means [56] for ‘time point’ were calculated to assess specific tSCS-induced short-term changes in outcome measures of the study.

To further investigate the influence of the grid montage on tSCS-induced changes, we expanded the linear mixed-effects model to assess interaction effects across two active stimulation conditions. This model included fixed effects for the interaction between the ‘time point’ (Pre, Post) and ‘stimulation condition’ (TRS and DIAG), capturing the dynamics of these interactions over time. The dependent variables (AUC, duration, and latency) were modeled as functions of the interaction term ‘time point *X* stimulation condition’ to investigate the grid montage dependence of the reflex responses. Random intercepts for ‘subject ID’ were included to account for individual variability across subjects, with random slopes for ‘time point’ to capture individual differences in response to the grid tSCS. Statistical significance was defined as *p* < 0.05 in all tests.

### 2.6. Qualifying experiments

We conducted a series of qualifying experiments with six participants to further explore the potential underlying mechanisms of grid tSCS neuromodulation. Five of the participants were involved in the original data collection and one new subject was recruited. For two participants, testing was performed on the left leg due to a history of right lower-limb injury occurring after the main experiment and within the past six months. These experiments aimed to assess whether tSCS was indeed applied at the sub-motor threshold and to explore the potential contribution of cutaneous afferent activation in the observed effects.

First, we assessed whether the grid tSCS was applied at the sub-motor threshold using a procedure adapted from posterior root muscle (PRM) reflex testing [57]. Single biphasic pulses (0.1 ms) were delivered via a current stimulator (DS5, Digitimer, Welwyn Garden City, UK), with stimulation intensity starting at 5 mA and increasing incrementally by 5 mA, up to 50 mA (a 25% higher than the actual amplitude used in the main experiment). Stimulation was distributed across eight channels through a splitter box as in the main experiment. The criteria of a motor threshold followed our prior definition (the lowest stimulation intensity that elicited a peak-to-peak amplitude of at least 100 *µ*V on TA [15]). Once the motor threshold was identified, 10 trials were acquired at 100% of the motor threshold to ensure a consistent response. A double-pulse stimulation protocol with interstimulus intervals set at 50 ms was employed. Both single and double pulses were delivered with a 10 s interval between each pulse to avoid habituation of the circuits. These protocols were employed to verify that the sub-motor threshold tSCS intensity used in the main experiments did not directly activate, nor transsynaptically activate, the efferent motor pathways.

Next, to assess the contribution of preferential recruitment of cutaneous afferents to the tSCS-induced changes, we conducted two experimental paradigms. First, we applied tSCS to the scapular region, targeting cutaneous receptors distant from the lumbar spinal segments innervating the TA muscles. This protocol also tests the spatial effect of our stimulation parameters.

Second, we explored the role of the onsite cutaneous afferents in the tSCS-induced changes by applying tSCS at the T10–T11 spinous process following topical application of 5% lidocaine to disrupt the transmission of signal between local cutaneous receptors and central nervous system [58]. The area corresponding to the grid location was treated with lidocaine after baseline TA flexion reflex responses were collected. The skin was exposed to lidocaine for 30 min, a duration suggested for an effective blockade of cutaneous receptors [59, 60]. To test the level of sensory reduction, we used three Semmens-Weinstein monofilaments (0.07 g, 0.4 g, and 2.0 g), chosen based on thresholds reported in the literature for sensory testing of thoracic and lumbar spinal segments [61]. The filaments were applied in a random order, with subjects blinded to the type of filament used. Sensory thresholds were determined when subjects reported detection in 2 out of 3 trials, with stimuli applied at random points within the tSCS grid area. Following lidocaine treatment, four out of six subjects exhibited increased sensory thresholds, indicating blockage of local cutaneous receptors. In two subjects, however, the sensory threshold remained unchanged at the 2.0 g filament.

Grid tSCS was then applied, and changes in the TA flexion reflex were recorded 30 min post-stimulation to determine whether blocking local cutaneous receptors would affect the neuromodulatory outcomes observed with tSCS.

Both ‘scapula’ and ‘lidocaine’ tSCS experiments were performed using DIAG montage, and the outcomes were evaluated in comparison to DIAG-induced changes in the same subjects.

## 3. Results

**Figure 4.**
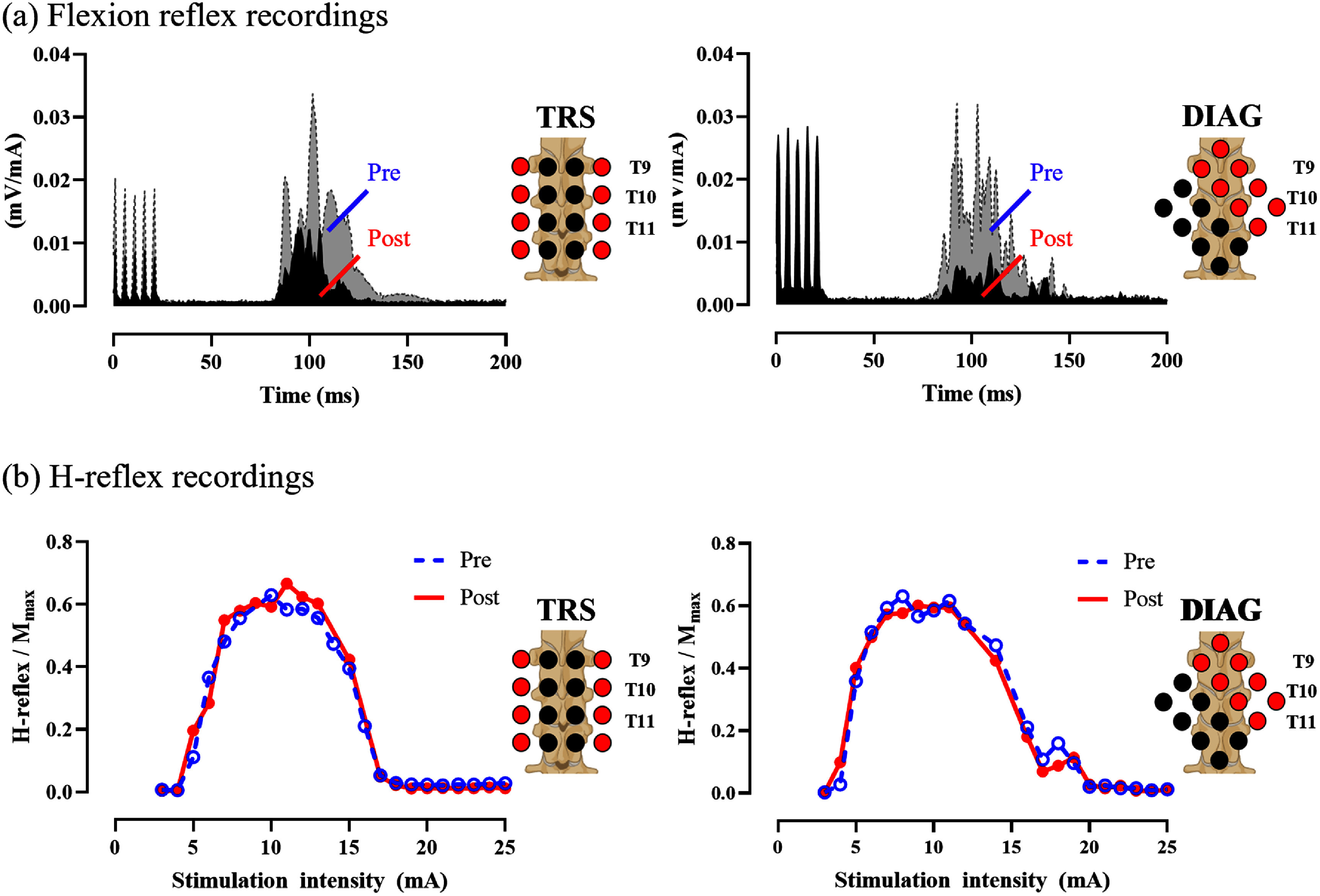
Reflex recordings in a representative subject (a) flexion reflex and H-reflex responses were obtained at Pre (dotted line), and Post (solid line) by TRS and DIAG grid tSCS in a representative subject. Flexion reflex amplitudes were normalized to the intensities of peripheral nerve stimulation (PNS) in milliamperes (mA). Both TRS and DIAG grid stimulation showing inhibition of the flexion reflex responses. (b) No discernible effect was observed on the H-reflex recruitment curve normalized to the peak M-wave. Created in BioRender. Dhaher, Y. (2025) https://BioRender.com/tyy4rgf.

**Figure 5.**
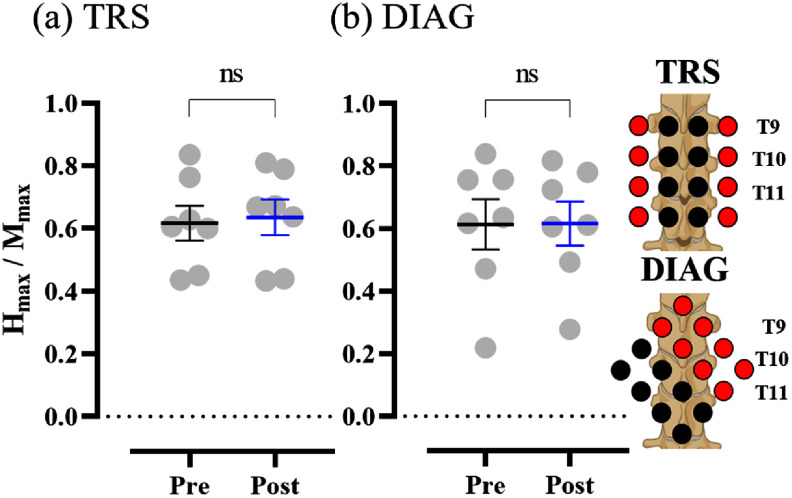
Effect of grid spinal stimulation on *H*-reflex The average *H*_max_/*M*_max_ ratio was evaluated at Pre and Post in a subcohort of 7 participants, using (a) TRS and (b) DIAG grid tSCS. No statistically significant changes were observed for either stimulation condition. Data are presented from 7 subjects from the total cohort. Created in BioRender. Dhaher, Y. (2025) https://BioRender.com/tyy4rgf.

Figure 4 shows a representative example of flexion reflexes and *H*-reflexes recorded from a single participant before and after active stimulation using two grid montages. These muscle reflex activities were exclusively observed in the TA and Sol muscles, corresponding to the acquisition of flexion reflex and *H*-reflex data. In this subject, both TRS and DIAG grid stimulations markedly decreased flexion reflex AUC (figure 4(a)), while no discernible effect was observed on the *H*-reflex recruitment curve normalized to the peak *M*-wave (figure 4(b)). The trend remained consistent when analyzing group-average responses from all study participants. The average *H*_max_/*M*_max_ ratio was assessed in a subcohort of 7 participants. Statistical analysis revealed no statistical difference in the ratio for either TRS [*F*(1, 5.31) = 0.020, *p* = 0.892] or the DIAG [*F*(1, 6.91) = 0.074, *p* = 0.794] stimulation conditions (figure 5).

**Figure 6.**
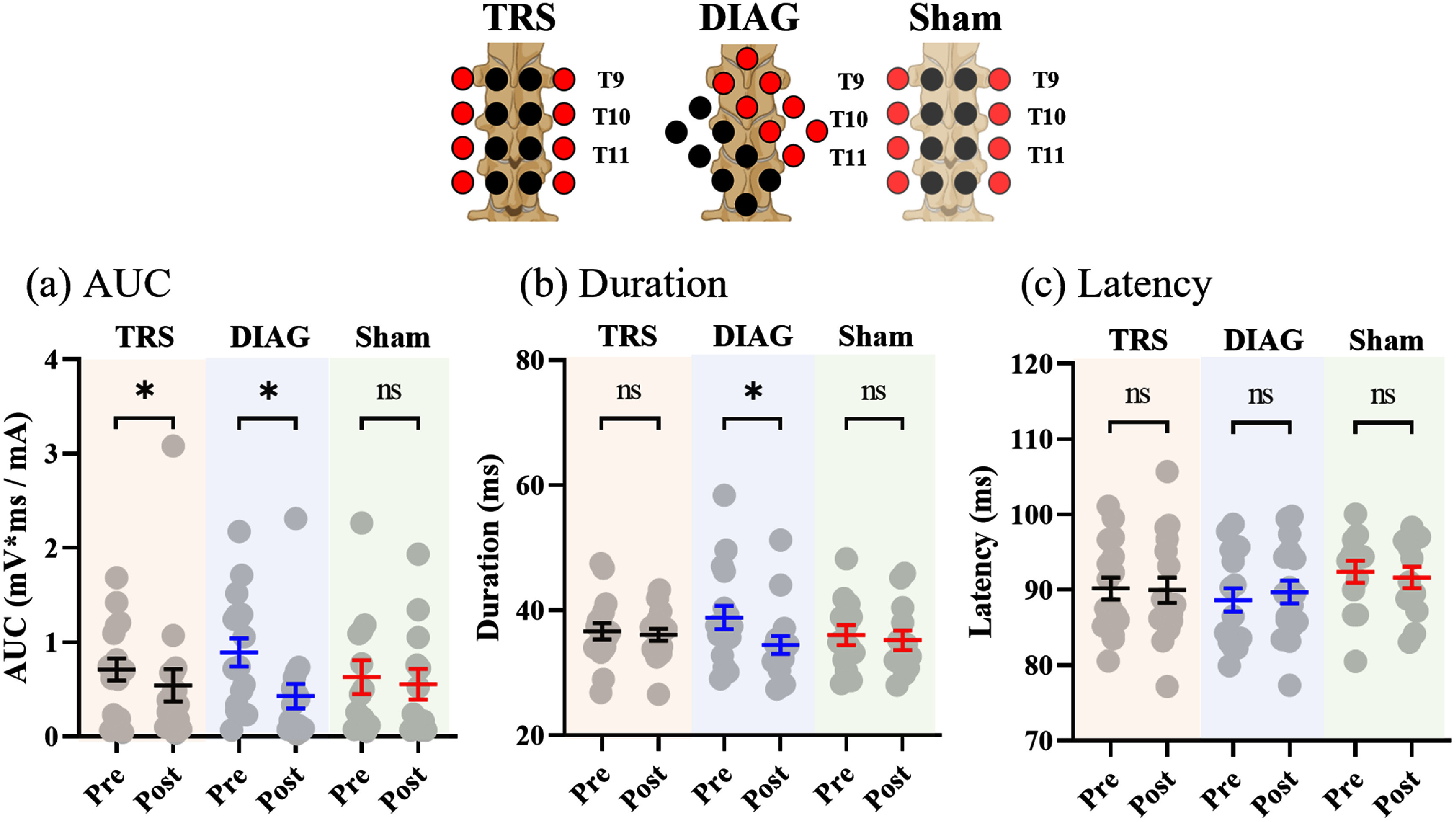
Effect of grid spinal stimulation on flexion reflex the effect of grid spinal stimulation on flexion reflex parameters were assessed for (a) AUC, (b) duration, and (c) latency across 17 subjects. Statistical analysis revealed significant changes in AUC for both TRS and DIAG conditions, while only the DIAG condition showed a significant change in duration. No statistically significant changes were observed in any outcomes for the sham condition, based on a subset of 13 subjects from the total cohort. Created in BioRender. Dhaher, Y. (2025) https://BioRender.com/tyy4rgf.

The application of tSCS induced a net inhibitory effect on the TA flexion reflex. The analysis of 17 participants indicated that both TRS and DIAG showed significant effects of time point on AUC for TRS [*F*(1, 16.5) = 13.2, *p* = 0.002] and DIAG [*F*(1, 16.2) = 10.7, *p* = 0.005] conditions, respectively. No significant difference was observed in the sham stimulation condition [*F*(1, 10.6) = 0.952, *p* = 0.351] (figure 6(a)).

In DIAG case, the observed decrease in AUC was accompanied by a reduction in duration [*F*(1, 16.4) = 8.95, *p*= 0.008]. However, a significant reduction in duration was not observed in the TRS condition [*F*(1, 16.6) = 0.08, *p* = 0.781] and sham condition [*F*(1, 296) = 0.191, *p* = 0.663] as shown in figure 6(b).

Across all stimulation conditions, no significant differences in reflex latency were observed (figure 6(c)). Specifically, latency remained unchanged for the TRS [*F*(1, 14.6) = 0.396, *p* = 0.539], DIAG [*F*(1, 16.8) = 3.22, *p* = 0.091], or Sham condition [*F*(1, 12.3) = 1.01, *p* = 0.335], while the DIAG condition showed a trend toward increased latency.

**Figure 7.**
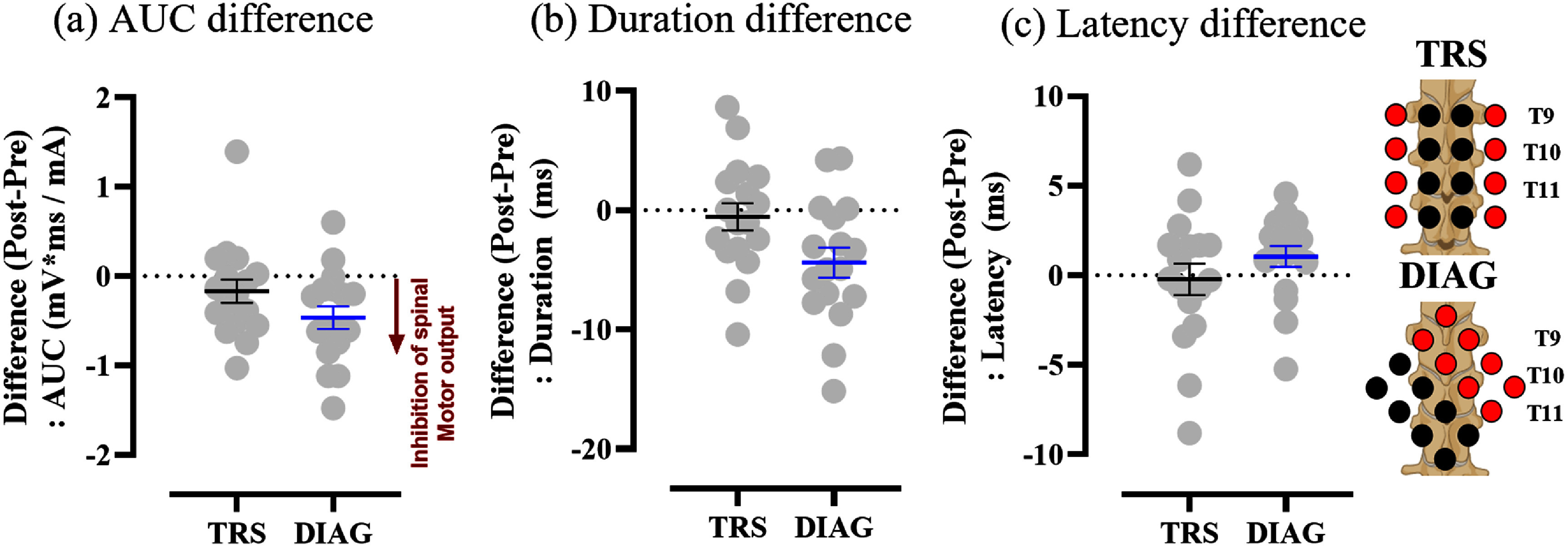
Grid montage-specific effects on flexion reflex data for each active stimulation condition are presented as mean difference (Post-Pre) ± SEM for (a) AUC (b) duration and (c) latency of flexion reflex across 17 subjects, comparing TRS and DIAG grid montages. Negative values in AUC and duration indicate inhibition of spinal motor output. Created in BioRender. Dhaher, Y. (2025) https://BioRender.com/tyy4rgf.

In the analysis of the interaction between stimulation conditions and time points, although the AUC remained consistent [*F*(1, 812) = 1.104, *p* = 0.294], significant effects were observed in duration [*F*(1, 818) = 6.02, *p* = 0.014], indicating that changes in duration are not uniform and vary by stimulation conditions. This aligns with our findings that showed a significant effect on reflex duration only in DIAG conditions. Additionally, significant effects were observed in latency [*F*(1, 816) = 7.09, *p* = 0.008]. The observed significance in latency can be explained by a trend toward delayed latency (*p* = 0.091) observed specifically in the DIAG condition. The group-averaged mean differences (Post-Pre; ± SEM) in AUC, duration, and latency for each active stimulation condition are shown in figure 7, providing a visual representation of the tSCS-induced changes. Specifically, the difference in AUC for TRS and DIAG were −0.17 ± 0.13 and −0.46 ± 0.13 (mV*ms mA^−1^), respectively (figure 7(a)). For duration, the differences were −0.54 ± 1.14 ms (TRS) and −4.38 ± 1.26 ms (DIAG) (figure 7(b)). The difference in latency were −0.22 ± 0.88 ms (TRS) and 1.05± 0.58 ms (DIAG) (figure 7(c)).

These findings suggest that different stimulation conditions distinctly affect the short-term changes in the TA flexion reflex outcomes, highlighting the differential neuromodulatory responses elicited by each grid montage.

### 3.1. Result: qualifying experiments

The PRM protocol confirmed that the tSCS intensity applied at the T10-T11 spinous process in this study was a sub-motor threshold, as no motor responses were elicited in the measured lower limbs even at 50 mA, an intensity higher than what was used in the main experiments (supplementary figure 1).

When tSCS was applied to the scapular region, no significant changes were observed in the TA flexion reflex for AUC [*F*(1, 110) = 2.83, *p* = 0.095], duration [*F*(1, 115) = 3.70, *p* = 0.057], or latency [*F*(1, 3.23) = 0.232, *p* = 0.661] (supplementary table 2), indicating that stimulating cutaneous afferents alone was insufficient to significantly modulate the reflex and that stimulation applied at a distant location does not affect the circuit excitability. It remains to be seen if an increase in the number of samples for this qualifying experiment will achieve significance for AUC (*p* = 0.095) and duration (*p* = 0.057). Nevertheless, when statistically examined, the trending level of inhibition observed with scapular stimulation, while statistically insignificant, was significantly smaller in AUC difference compared to when the grid was applied over the site of interest on the spine for the DIAG configuration [*F*(1, 237) = 12.6, *p* < 0.001], with a trend toward significance in latency [*F*(1, 235) = 3.125, *p* = 0.078]. This suggests that while scapular stimulation had some inhibitory effects, they were less pronounced than those observed with spinal (DIAG) stimulation.

Finally, applying tSCS at the T10–T11 spinous process after the cutaneous block using the 5% lidocaine yielded an inhibitory effect in the TA flexion reflex. The observed changes in AUC were statistically significant [*F*(1, 5.07) = 9.55, *p* = 0.027], while in duration [*F*(1, 10.0) = 2.51, *p* = 0.144] and latency [*F*(1, 4.83) = 0.885, *p* = 0.392] was not (supplementary table 2). These findings suggest that the neuromodulatory changes were not solely dependent on local cutaneous afferent input, indicating the involvement of other spinal circuit components. Additionally, the observed inhibition in tSCS with lidocaine was not statistically different from DIAG configuration in AUC difference [*F*(1, 265) = 0.005, *p* = 0.944], in duration [*F*(1, 268) = 1.408, *p* = 0.236] and in latency [*F*(1, 265) = 1.351, *p* = 0.246]. Trends in group-averaged differences (Post-Pre; ±SEM) for AUC, duration, and latency between the scapula, lidocaine, and DIAG conditions are presented in supplementary figure 2.

## 4. Discussion

Our study aimed to investigate the modulatory effects of a multi-electrode tSCS paradigm on spinal reflex excitability, with a particular interest in short-term neuromodulatory effects. We implemented a stimulation paradigm using a grid of electrodes with montages designed to create a net-applied electric field in the anatomic transverse and diagonal directions to the lumbar spinal segment. We utilized the well-established flexion reflex paradigms and *H*-reflex paradigms to assess the neuromodulatory changes in polysynaptic spinal neuronal excitability and motoneuron excitability, delineating potential plasticity that may occur within the spinal neural pathway.

Our primary hypothesis was that the grid tSCS could induce short-term changes in spinal circuit excitability and that the observed plasticity depends on the grid electrodes’ specific montage due to the electric field’s varying spatial distribution. Specifically, the DIAG montage, which generated a greater electric field in all orientations (supplementary figure 3), was expected to produce a more pronounced neuromodulatory effect.

### 4.1. Neuromodulatory effects of grid tSCS

Our grid tSCS paradigm demonstrated modulation of spinal neural circuits 30 min after cessation of tSCS, resulting in a net inhibitory effect in TA flexion reflex measure (AUC) for both grid montages. Additionally, while the latency of the flexion reflex remained consistent across all stimulation conditions, an increasing trend was observed specifically in the DIAG condition. Notably, the inhibitory effect in the AUC was accompanied by a significant reduction in reflex duration only in the DIAG condition, suggesting that grid montage would likely have a differential effect on the stimulation induced neuromodulation.

While prior studies report immediate inhibitory effects during the application of tSCS [62, 63], our findings demonstrate that these inhibitory effects may persist well beyond the cessation of stimulation. This aligns with previous studies showing sustained inhibitory effects of tSCS in post-SCI spasticity [13, 64] and abnormal nociceptive withdrawal reflex [21]. Our study further demonstrates that tSCS can produce lasting modulation in the spinal cord, with grid-specific effects on spinal neuronal excitability.

In the sham stimulation condition, no statistical significance was observed in the TA flexion reflex outcome measures (figure 6). The lack of significance in sham conditions may be partly due to a smaller sample size (*n* = 13) than in active tSCS conditions (*n* = 17). However, the significance observed in the DIAG and TRS conditions remained consistent when statistical analyses were conducted only in 13 participants.

The neuromodulatory effect on the Sol *H*_max_/*M*_max_ ratio did not reach statistical significance for either grid. The lack of significant change in the *H*_max_/*M*_max_ ratio suggests that tSCS may not substantially alter the maximal motoneuron pool recruitment, especially in healthy individuals. While other studies have reported changes in *H*-reflex parameters, such as homosynaptic depression [65], our findings in *H*_max_/*M*_max_ ratio align closely with previous examinations in healthy participants reporting no significant changes following tSCS [40, 66, 67].

### 4.2. Montage-specific effects of the grid tSCS

Our results also highlight the effect of grid configuration may have in modulating the spinal circuit excitability. The observed reduction in AUC in both TRS and DIAG conditions reflects changes in both the amplitude and duration of the TA flexion reflex, indicating an overall decrease in the excitability of the polysynaptic pathway [21]. This could suggest reduced excitatory synaptic input or increased inhibitory regulation within the spinal circuits, affecting both the magnitude and duration of the response.

The increased trend in latency observed in the DIAG condition could be attributed to changes in synaptic connectivity. Specifically, an increase in synaptic connections, along with a higher density of interneurons within cultured neuronal networks, has been associated with prolonged latencies [68]. Similarly, computational simulations employing Hodgkin–Huxley (HH) neuron models suggest that greater circuit complexity, characterized by the inclusion of additional neurons and extended branching patterns, can result in delayed response onset [69]. Furthermore, the latency trend observed in the DIAG condition may also reflect the recruitment of slower-conducting afferent fibers or the engagement of more intricate interneuronal pathways [70]. This finding suggests that the anode/cathode configuration not only influences neuronal excitability but may also modulate the excitability of distinct interneuronal circuits, linked likely to the changes in reflex duration.

The reduction in reflex duration observed in the DIAG condition suggests that motoneuron activity may be terminated more rapidly, potentially due to modified synaptic integration [71]. This could be attributed to faster termination of the excitatory input or enhanced recruitment of inhibitory interneurons [72], leading to a shorter overall response. Such findings suggest that the stimulation montage can lead to distinct effects on the temporal properties of the flexion reflex, facilitating future examination for targeted changes.

Employing our published high-fidelity electromagnetic field finite element model of the human trunk [51], we sought to assess the spatial extent of the grid-induced electrical field in neural structures at the spinal cord. In this simulation, we placed the grid over the targeted segment, emulating the experimental design. Both the TRS and DIAG montages used the methods outlined by Makarov *et al* [51] yielded an appreciable electrical field at the cord level with a stimulation amplitude of 40 mA. Our simulation showed that the DIAG montage generated a greater electric field magnitude than the TRS montage, exceeding the 0.15–0.18 V m^−1^ range linked to neuronal plasticity in experimental animal models [24]. Upon further examination of our estimation of the induced electrical field at the cord, the DIAG montage generated greater caudal–rostral, ventral–dorsal, and right–left components of the electric field compared to the TRS montage, suggesting a montage-specific impact on the spinal circuits aligned with these components (supplementary figures 3(a) and (b)). Moreover, the DIAG montage produced electric fields above 0.15 V m^−1^ across a broader spinal region (supplementary figure 4). On the other hand, the TRS montage exhibited a more localized electric field distribution, with key regions within the gray matter of the L4/L5 segments, particularly laminae III–IV (processing non-noxious stimuli) and V–VI (synaptic integration) [73], reaching the 0.15 V m^−1^ threshold in smaller areas. Together, this indicates that the DIAG montage may have greater neuromodulatory potential within spinal circuits.

Moreover, our analysis showed that the electric field generated by the TRS montage remained below the 0.15 V m^−1^ threshold in laminae IX of the S1/S2 segments (Sol motoneuron), while the DIAG montage produced fields exceeding this threshold. Regardless, no significant change was observed in the *H*_max_/*M*_max_ ratio, indicating that the induced electric field may not have directly influenced the monosynaptic pathway of Sol. This suggests that grid-based tSCS may primarily modulate spinal circuit components, with the observed neuromodulatory effects potentially linked to network-level changes within the spinal cord. However, our model does not definitively determine whether one montage would consistently increase or decrease neuronal activation, as the precise mechanisms involving neuron orientation and directional field effects are not yet fully understood. Future studies, including HH neuron models or biophysical neuron models of TA flexion reflex or Sol *H*-reflex, may provide better insight into grid-specific changes in the orientation of neurons and the electric field.

Additionally, the observed variations in response to the grid montage may be partially attributable to the anisotropic properties of the spinal cord, which influence the volume distribution of the induced electric field [74]. It is interesting to note that the observed grid-dependent change in flexion reflex opens the opportunities for a complete parametric study where a selected set of anode and cathode electrodes within the grid can be designed for targeted reshaping of the activities of interest, up-or down-regulation of the reflex activities. Such targeted stimulation construct can be informed by the high-fidelity finite element model [75] coupled with models of motoneurons and interneurons in the spinal cord [52].

The differential changes between TRS and DIAG montages suggest that our tSCS approach can elicit distinct post-stimulation effects. This finding provides a foundation for further research to explore the optimal configurations for tSCS, using larger and more uniform sample sizes to validate these preliminary observations.

### 4.3. Exploring potential contributors

The precise origin of the observed inhibitory effect remains unclear, especially considering the time window of the assessment—30 min post-grid stimulation offset. However, one possible explanation is that the changes in the flexion reflex resulted from stimulation-induced direct polarization of nerve roots [76, 77] and/or white matter tracts. Prior studies, however, indicate that the effects of stimulation-induced polarization of afferents and axons are transient [16, 78] lasting no longer than two minutes [79]. In contrast, our observed neuromodulation in the flexion reflex persisted for 30 min post-stimulation, well beyond the timeframe required for white matter tracts, including those in the dorsal column, to return to homeostasis [80]. These findings suggest that the observed reflex modulation is unlikely to be solely attributable to non-synaptic root polarization and may involve additional neuronal pathways.

To systematically evaluate and rule out other potential confounding factors, we conducted a series of qualifying experiments in a subset of participants. Specifically, we employed a PRM protocol with a DIAG montage, confirming that the 40 mA tSCS applied at the T10–T11 spinous process was subthreshold for motor activation, as evidenced by the absence of muscle responses in the lower limbs, even at an increased intensity of 50 mA. This finding effectively excludes the possibility of direct or transsynaptic activation of motor pathways.

Local cutaneous receptor blockade under the stimulation grid using 5% lidocaine did not alter the inhibition of the TA flexion reflex, indicating that cutaneous afferent activation was not likely the driver of the observed neuromodulation. A detailed analysis of the qualifying data revealed that only 16% of the observed inhibition could be attributed to cutaneous afferents. Furthermore, remote cutaneous afferent stimulation using an identical grid montage at 40 mA intensity, applied to the scapular region, did not elicit significant reflex changes, indicating that the observed effects were not generalized cutaneous responses. Secondary to the cutaneous assessment, the absence of inhibition under scapular stimulation further supports the localized nature of our stimulation paradigm. Collectively, these findings suggest the involvement of the cutaneous response to the short-term plasticity of our stimulation is unlikely.

Although superficial cutaneous afferents were unlikely contributors to the observed short-term plastic effect, the potential contribution of root axons associated with sensory afferents cannot be entirely excluded. Biophysical simulations suggest that a subset of large-diameter A*β* fibers (12 *µ*m) can be activated at approximately 35 mA [81], though this constitutes only a small fraction of the total A*β* population at T10–T11 [82]. The model further indicates that, on average, A*β* fibers require higher currents (∼70 mA) for activation, suggesting that our 40 mA stimulation likely resulted in only limited activation of these fibers. In addition, while A*α* fibers can be activated at 40 mA, the absence of significant *H*-reflex changes 30 min post-stimulation suggests minimal impact on the monosynaptic pathway. Additionally, recent studies in animal models indicate that the polarization of nociceptive C-fibers (without concurrent fiber activity) can lead to transient excitability changes [83]. Given the short latency of the polysynaptic reflex observed in this study (60–100 ms), it is highly unlikely that C-fibers contributed to the observed response, as the reported latency for C-fiber responses at the human ankle exceeds 145 ms [84]. Further investigations are warranted to determine whether the polarization of A*β* fibers in animal models (in the absence of concurrent fiber activity) could similarly induce short-term excitability changes.

The mechanisms underlying neuronal plasticity induced by transcutaneous spinal electrical stimulation remain an area of active debate. However, evidence suggests that the neuromodulatory effects of stimulation arise from a combination of multiple mechanisms, including spinal interneuron modulation, spike-timing-dependent plasticity-Hebbian-like plasticity, reinforcement of corticospinal projections, alterations in motor cortex excitability, neurochemical modulation (likely via serotonergic and dopaminergic pathways), and reflex pathway reorganization [16, 85–89].

One plausible mechanism for the sustained inhibition observed in this study involves stimulation-induced synaptic plasticity within spinal circuits, potentially mediated by long-term potentiation or long-term depression [90]. These synaptic modifications may alter spinal transmission gain by modulating interneuronal network activity [27]. Indeed, long-lasting modifications in motoneuron firing properties following trans-spinal direct current stimulation have been reported in rats [91]. The involvement of these inhibitory and excitatory interneurons, along with their associated motoneurons, likely represent key sites of neuromodulation during tSCS, ultimately influencing the spinal motor output observed in this study.

Specifically, polysynaptic pathways associated with A*β*/*δ* afferent fibers, and their interneurons may contribute to this modulation [92]. Animal studies have identified interneurons that receive direct A*β* and A*δ* afferent inputs and project to the dorsal and intermediate spinal cord regions [93–95]. Notably, ROR*α* (excitatory) and ROR*β* (inhibitory) interneurons are key components of sensorimotor circuits involved in polysynaptic reflex pathways linked to A*β*/*δ* fibers. These interneurons process sensory input from mechanoreceptors and modulate ipsilateral alpha motoneuron activity. Research by Bui *et al* has further identified dl3 interneurons, which function similarly to ROR*α*, receiving input from low-threshold mechanoreceptors (A*β*) and projecting to ipsilateral motoneurons [93]. Polysynaptic reflexes, mediated by interneuronal networks within the spinal cord, have been suggested to fluctuate with the functional state of spinal circuits. Prior research suggests that modifications in polysynaptic reflex properties serve as an indirect marker of neurophysiological plasticity within the spinal network [96].

Our data indicates that grid-patterned stimulation elicited measurable changes in polysynaptic reflex duration and amplitude, observed 30 min post-stimulation offset. It is plausible that the subthreshold tSCS employed in this study modulated the physiological state of both interneuronal and motoneuronal membranes associated with A*β*/*δ* afferent pathways, independent of direct afferent activation. This interpretation aligns with the principles of activity-independent plasticity, wherein sustained alterations in neuronal properties emerge in the absence of overt synaptic activity [81].

The selected anode–cathode montage yielded short-term inhibition of the polysynaptic reflex. Previous research in rats has demonstrated a prolonged reduction in the spontaneous firing of wide dynamic range neurons in the dorsal horn following sub-motor threshold spinal stimulation [85]. A proposed mechanism for sustained inhibition involves modulation of GABAergic synapses and NMDA receptor activity [97, 98]. This modulation may lead to presynaptic inhibition [99] and an increase in intracellular calcium levels in postsynaptic neurons, potentially inducing enduring changes in neuronal excitability. Although NMDA receptor activation typically requires synaptic input, studies have shown that receptor activity can be modulated even in the absence of strong afferent drive [97].

Electrical stimulation may have also directly influenced cortical excitability via ascending spinal pathways, including the spinothalamic and spinoreticular tracts. This modulation could enhance cortical motor neuron recruitment, thereby strengthening motor output to spinal circuits. Notably, the stimulation paradigms used in this study resulted in inhibition rather than excitation. Nevertheless, further investigations are warranted to monitor brain activity, particularly 30 min post-stimulation. Additionally, stimulation-induced neuromodulator expressions likely contribute to the observed reflex changes. To elucidate this relationship, concurrent measurements of serum serotonin, dopamine, and other neuromodulators alongside electrophysiological assessments are recommended.

In summary, the findings of this study suggest that transcutaneous spinal electrical stimulation induces long-lasting neuromodulatory effects through multiple mechanisms, including spinal interneuron modulation, synaptic plasticity, and neurochemical regulation. The observed inhibition of the polysynaptic reflex, in the absence of direct motor pathway activation, highlights the complexity of spinal network interactions. Future investigations should aim to further elucidate the underlying pathways, particularly through concurrent neurochemical and electrophysiological measurements, to refine our understanding of spinal plasticity and its potential applications in neuromodulation therapies.

### 4.4. Practical considerations

The reliability and validity of the TA flexion reflex as a measure of spinal excitability have been established in previous research, indicating reliability and reproducibility [100, 101]. Although studies focusing exclusively on the A*β*-fiber-mediated flexion reflex are limited, existing evidence shows that this response can be reliably elicited at low-intensity (sub-pain) [45, 102]. The TA flexion reflex has proven applicability for evaluating spinal circuit modulation under various conditions, including rest [21] and during walking [31, 103, 104]. These studies support our use of the TA flexion reflex as the primary outcome in this study, providing a foundation for assessing spinal excitability modulation by grid tSCS.

Given the inherent variability of the anatomical structures and the neurophysiology of spinal circuits across individuals, a standardized approach for determining stimulation intensity is crucial to comprehend the relationship between the stimulation dose (input) and the stimulation-induced modulations in the reflex responses. Factors such as the distance between the spinal cord and skin and the volume and thickness of other underlying structures could influence the resulting electric field generated by the spinal stimulation in each participant [74]. This suggests that spinal circuits may have been exposed to varying electric fields across subjects. Therefore, individualizing the stimulation intensity and precisely positioning the grid for each participant could provide a better understanding of the stimulation effects.

Several studies have identified the optimal electrode locations by delivering single or double stimuli through the electrode applied to the cord, monitoring for the site that elicits the strongest sensory or motor response in the target muscle [12, 57]. Furthermore, sensory or motor thresholds were systematically identified by raising the intensities in 1–5 mA increments up to each participant’s tolerance level or a percentage of the motor threshold [28, 105–107]. Due to the proof-of-concept nature of the current study, a fixed level of electric current was used across subjects. Future examinations should explore a systematic approach to individualizing stimulation intensity and electrode location to ensure more reliable results across participants.

Moreover, no standardized guidelines currently inform the choice of stimulation parameters specifically for modulating spinal neural excitability related to the TA flexion reflex and observing effects 30 min post-stimulation. Clinical efforts to modulate lower motor functions have varied widely, with monophasic or biphasic pulse frequencies ranging from 30 to 50 Hz and stimulation durations from 5 to 45 min, with 20 min emerging as the most common duration. While some studies have employed high intensities above 100 mA [105, 108], most have utilized sub-motor or motor threshold intensities, depending on the application [11, 57, 105].

Given the exploratory nature of our study, our selection of 100 Hz and sub-motor threshold intensity (5 mA per electrode) aimed to examine the neuromodulatory impact on spinal neural excitability, anticipating this could contribute to establishing more refined protocols in future research. Recalling that one of our primary goals was to test if the neuromodulator effect of stimulation is likely linked to the spatial distribution of the applied electrical field. In this context, the choice of frequency is likely less detrimental to this goal.

In this study, we aimed to enhance the stimulation-induced effect using a localized stimulation approach with a spatially confined grid of electrodes, incorporating both anodes and cathodes. Although the efficacy of targeting specific spinal segments in epidural stimulation cannot be achieved in tSCS, we sought to enhance the stimulation-induced effects through this grid stimulation approach. However, the implementation of small electrodes for localized tSCS poses several challenges. A potential concern is whether a spatially confined grid may not generate an electric field strong enough to reach the spinal cord effectively. Our recent study addressed this concern through a simulation using a high-fidelity human spinal cord model [75]. The results demonstrated that the electric field covers narrower sections of the spinal cord with the electrical potential in the gray and white matter well above 0.15 V m^−1^ (supplementary figure 5), an assumed minimum threshold value for effective neuromodulation [23, 34, 35]. These findings align with our experimental results, demonstrating significant neuromodulatory effects on reflex response through spinal stimulation.

While our primary target for neuromodulation was the TA (the grid placed on the T10–T11 spinous process), we did not explicitly assess the excitability of group Ia monosynaptic pathway (hence the motoneuron excitability) of the TA due to methodological difficulties in eliciting *H*-reflex in TA muscle, especially in healthy participants. Alternatively, we tested the potential effect of the grid stimulation in the adjacent motoneuron pool excitability, motoneurons involved in the activation of the Sol muscle. However, this approach has limitations due to differences in the neural circuitry and spinal segmental innervation of the two muscles. The TA and Sol muscles are innervated by different spinal segments- Sol by the S1–S2 segment and TA by the L4–L5 segments [37]. As a result, grid tSCS applied at the T10–T11 level may affect the motoneuron pools differently, and the applied electric field may not influence the motoneurons in the Sol as effectively as those in the TA. The later observation, combined with the lack of changes in the *H*-reflex of the Sol muscle, provides indirect evidence of the focal nature of the proposed stimulation construct. Future studies that evaluate the monosynaptic excitability of the TA could provide a better assessment of how grid stimulation impacts motoneuron-specific excitability.

### 4.5. Clinical significance of the Aβ fiber pathways

Prior work has highlighted the fundamental role of spinal reflexes, including A*ß*-fiber mediated flexion reflexes, in the regulation of human movement [109, 110]. These reflexes undergo significant alterations across a range of pathological conditions. For instance, research by Zehr and Loadman demonstrated diminished phase-dependent modulation of flexion reflexes during gait (A*ß* type) in patients with SCI and stroke [111]. Other pathological indicators include prolonged muscle reflex activities, hyperreflexia in the monosynaptic pathway (e.g. *H*-reflex), and the emergence of abnormal polysynaptic reflexes, including A*ß*-fiber driven reflexes (e.g. Babinski reflex) [109]. The extent to which these alterations in the reflex pathway contribute to gait dysfunction remain largely unknown. However, a prevailing hypothesis suggests these alterations arise from impaired supraspinal descending control over the spinal network, mainly due to a lack of descending inhibitory control within the central nervous system. Despite the differences in the neurophysiology of the polysynaptic reflex circuit between healthy individuals and those with SCI or stroke [112, 113], the observed inhibitory effect of tSCS on reflex circuit responses suggests the potential to improve the altered function of A*ß*-fiber mediated flexion reflexes in these pathological states.

Furthermore, studies by Wagner *et al* [14], Peña Pino *et al* [114], and Rejc *et al* [115] have shown that epidural stimulation can improve motor function after stimulation offset, often involving modulation of spinal reflex properties [31, 116]. If non-invasive stimulation can yield results on par with invasive stimulation in motor rehabilitation, it would benefit patients by eliminating the need for surgical procedures and their associated recovery period. The guided noninvasive spinal cord stimulation design proposed herein could be a viable tool to enhance outcomes in targeted motor training or rehabilitation scenarios.

## 5. Conclusion

Our study has demonstrated the capability to induce short-term inhibition on spinal circuits through a brief period of tSCS. This outcome presents potential advantages for helping individuals regain lost motor functions. Such a discovery is promising for clinicians and patients seeking more targeted and effective rehabilitation strategies by leveraging a multi-electrode tSCS to reshape the neurophysiological properties of the spinal circuits.

## Data Availability

The data for the study has been archived and requires significant effort to physically locate and recover. The data that support the findings of this study are available upon reasonable request from the authors.
